# Heterotopic Triplet Pregnancy after In Vitro Fertilization with Favorable Outcome of the Intrauterine Twin Pregnancy Subsequent to Surgical Treatment of the Tubal Pregnancy

**DOI:** 10.1155/2014/356131

**Published:** 2014-01-12

**Authors:** Theodoros Felekis, Christodoulos Akrivis, Panagiotis Tsirkas, Ioannis Korkontzelos

**Affiliations:** Department of Obstetrics and Gynecology, “G. Hatzikosta” General State Hospital, Makrygianni Avenue, 45445 Ioannina, Greece

## Abstract

Heterotopic triplet pregnancy is an exceptionally rare medical condition. The broad use of assisted reproductive technologies has contributed to the increase of ectopic and subsequently heterotopic pregnancy rate, masking a life-threatening condition for the gravid and the intrauterine pregnancy. We describe a case of a woman with heterotopic triplets at 9^+4^ gestational week following transfer of three embryos obtained by in vitro fertilization techniques. The ectopic tubal pregnancy was ruptured and salpingectomy was performed by laparotomy. The intrauterine pregnancy progressed to the delivery by cesarean section of two healthy twins at 36^+2^ gestational age. Heterotopic triplets with tubal ectopic are a special diagnostic and therapeutic challenge for the obstetrician. High index of suspicion and timely treatment by laparotomy or laparoscopy can preserve the intrauterine gestation with a successful outcome of the pregnancy.

## 1. Introduction

Heterotopic pregnancy (HP) is a rare medical condition in obstetrics. It is characterized by the presence of coexistent intrauterine and ectopic pregnancy. The most frequent implantation site of the ectopic pregnancy is in the fallopian tube, most commonly in its ampullary segment (80%) [1]. The incidence of heterotopic pregnancy is around 1/30,000 (1/10000 to 1/50000) in spontaneous pregnancies [1, 10]. In pregnancies resulting from assisted reproduction techniques, the incidence is greater, ranging from 1/100 to 1/3,600, nearly as high as 1% in some series [1]. Overall, the incidence of heterotopic pregnancy nowadays is estimated around 1/7000 [2] to 1/15000 live births (0,8% calculated risk) in contrast with the indisputably lower incidence of 1 : 30.000 in 1948 [3]. The big difference in these percentages is attributed to the higher incidence of pelvic inflammatory disease (PID) observed currently resulting in tubal damage as well as ovarian stimulation and transfer of many embryos in the range of the broad use of assisted reproductive technology (ART) techniques [1]. There has also been a 5.9-fold increase in triplet conception between 1971–1977 and 1998, attributable to ART extended use, too [4]. Heterotopic triplets are even more uncommon and cases with tubal ectopic and coexisting twin intrauterine pregnancy are limited. This medical condition can be hazardous to the intrauterine pregnancy and mother's life as well. We present the case of a following in vitro fertilization (IVF) combined intrauterine twin and tubal pregnancy which was ruptured and successfully surgically treated with preservation of the intrauterine pregnancy. The final outcome was the birth of healthy twins. The aim of this paper is to emphasize the need for raised clinical suspicion of this clinical entity during the routine first trimester ultrasound examination, even in the presence of an intrauterine multiple gestation and especially when predisposing factors like IVF are present.

## 2. A Case Report

**Figure 1:**
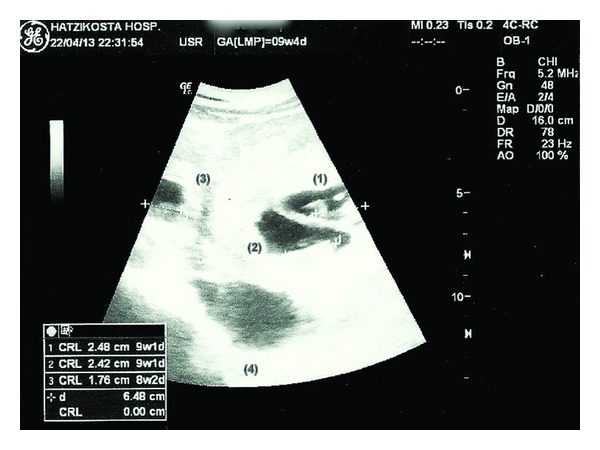
Ultrasound examination shows an intrauterine twin pregnancy (1, 2) with crown-rump length measurements of 24,8 (1) and 24,2 (2) mm according to 9^+1^ weeks, a third gestational sac in the right uterine adnexa (3) with an embryo measuring 17,6 mm length and free fluid in the pouch of Douglas (4).

A 36-year-old woman (gravida 2, para 0) with a confirmed intrauterine twin pregnancy after her first attempt of in vitro fertilization and implantation of three embryos was transferred by ambulance to the emergency department of our hospital from a regional hospital, where she had been hospitalized for 24 hours for suspected ovarian hyperstimulation syndrome. She was at 9^+4^ week of gestation and presented with gradually worsening right lower abdominal pain referring to the back, ribs, and the tip of the right shoulder, with nausea and vomiting. Her medical record was free apart from a spontaneous first trimester miscarriage and had a three-year history of infertility with no obvious underlying factor. On admission she was pale and cold, with a normal level of consciousness. Pulse rate was 102/min, and blood pressure was 80/60 mmHg. Hemoglobin and hematocrit were 7.3 g/dL and 21,4%, respectively, white blood cell count was 15.200/uL, and serum hCG was 366180.1 mIU/mL. Platelets count and prothrombin (PT) and activated partial thromboplastin time (aPTT) were within normal limits. Physical examination demonstrated marked right adnexal tenderness and diffuse abdominal and rebound tenderness. Ultrasound examination demonstrated a viable intrauterine twin pregnancy with crown-rump length measurements of 24,8 and 24,2 mm according to 9^+1^ weeks, a third gestational sac in the right uterine adnexa with an embryo measuring 17,6 mm length without cardiac activity, and the presence of free fluid in the pouch of Douglas and the abdominal cavity (Figure 1). She was immediately transferred to the operating room and underwent an emergency laparotomy, where a large hemoperitoneum was encountered. A ruptured right tubal ectopic pregnancy was confirmed and a right salpingectomy was performed while the patient received blood and plasma transfusion. The patient's postoperative recovery was uneventful and she was discharged on the fifth day. Histopathological examination verified the diagnosis.

The obstetric follow-up and fetal assessment were normal with good fetal growth of each twin. The patient had an uneventful pregnancy course until she had contractions and at 31^+6^ weeks of gestation and was admitted to the hospital due to a threatened preterm labor. She received tocolytics (atosiban and ritodrine) and corticosteroids (betamethasone 12 mg, two doses 1 day apart, intramuscular) in case of a future preterm delivery. Her hospitalization was continued until 36^+2^ weeks of gestation when a cesarean section was performed due to cervical effacement and contractions. The dichorionic diamniotic neonates were females, with birth weights of 2460 and 2600 g, respectively, and admission to the neonatal unit was not required. Postpartum course was unremarkable for the mother as well.

## 3. Discussion

Heterotopic triplets are rarely encountered in everyday clinical practice. However, the extended use of ART procedures nowadays has increased the ectopic and subsequently the heterotopic pregnancy (HP) rates. This clinical entity represents a potentially life-threatening condition for the woman and the intrauterine pregnancy. Factors predisposing to HP are identical to those predisposing to ectopic pregnancy: factors related to IVF like large number of transferred embryos, a transfer near the uterine horn, excessive pressure on the syringe and deep insertion of the catheter during transfer, the quality of the embryos, the hormonal milieu at the moment of transfer, the use of gonadotropins, the amount of fluid used as media for the embryos, and also adhesions related or not related to endometriosis and pelvic inflammatory disease (PID) [5, 6]. In our case the reported information is scarce. The implantation of an embryo in the wall of the fallopian tube bears a high risk of rupture because of the rich local vascularization and trophoblast invasion may cause tubal rupture even if there is no fetal cardiac activity. In cases of heterotopic pregnancy following IVF the diagnosis can be exceptionally difficult. The *β* chorionic gonadotropin (*β*-hCG) may continue to rise normally, the ovaries present enlarged, the ectopic gestational sac can easily be missed on ultrasound scan, and the intermittent unilateral pain can be attributed to a haemorrhagic corpus luteum or ovarian hyperstimulation—like in our case [7]. It is reported that approximately 70% of heterotopic pregnancies are diagnosed between 5 and 8 weeks of gestation, 20% are diagnosed between 9 and 10 weeks, and the remaining 10% are diagnosed after 11 weeks [8]. Only in 57% of cases presented in the literature the diagnosis of heterotopic triplets was preoperatively made [9]. Around 50% of heterotopic pregnancies are asymptomatic [10]. Most of them (78.5%) were diagnosed after the rupture of the tube, with acute abdomen symptoms [9]. Abdominal pain due to peritoneal irritation is the most frequent symptom appearing in 82.7% of heterotopic pregnancies. The extended use of transvaginal sonography nowadays has increased the preoperative diagnosis—even before the rupture. On routine ultrasonography the obstetrician should search the adnexa for a possible concurrent ectopic pregnancy, especially in case of an acute abdomen, even in the presence of an intrauterine multiple pregnancy. Index of suspicion should be higher in women with risk factors. Finally, the diagnosis should not be missed when a woman with a known intrauterine pregnancy presents with abdominal pain due to peritoneal irritation, hemoperitoneum, and hypovolemic shock as the woman of our presented case. The purpose of the treatment is to interrupt the development of the ectopic pregnancy and preserve the intrauterine pregnancy. The therapeutic options vary. Most cases of HP with tubal pregnancy have been treated surgically. The most frequently described treatment is surgical, by resection of the uterine horn (salpingectomy) by laparotomy or laparoscopy. Traditionally, laparoscopic treatment has been used to treat unruptured ectopic pregnancies at a small gestational age, while laparotomy has been used for ruptured ectopic [9, 11]. Although long-term effects of laparoscopic surgery during pregnancy on the fetus have not been well studied [9, 12], laparoscopy has been increasingly used in surgical procedures in pregnant women and according to the literature no increase in adverse outcomes has been reported [9, 13, 14]. Conservative treatment by locally injecting potassium chloride or hyperosmolar glucose is an option in HP, especially in cases involving cervical pregnancy [5, 8, 15]. The use of methotrexate has detrimental effects on the residual intrauterine pregnancy and is not an alternative [8, 9]. A review of the literature by Goldstein et al. revealed that 55% of tubal heterotopic pregnancies treated by KCl injection required subsequent salpingectomy which disputes the suitability of the method [16]. During the surgery the uterus should be minimally manipulated to prevent contractions during or after the operation. Nevertheless, special attention should be paid to prevent the disruption of the ovarian blood supply, particularly in the ovary bearing the corpus luteum. In case of disturbance of the corpus luteum up to 12 weeks of gestation, the progesterone support is indicated [9]. In our case, we proceeded immediately to resection of the ruptured salpinx by laparotomy, after the diagnosis was established by clinical and ultrasound criteria. A laparoscopic approach did not present as an option due to the presence of hemoperitoneum and the hemodynamic instability of the patient.

The prognosis of the intrauterine gestation after treatment of the ectopic pregnancy is good. The rate of live births in heterotopic triplets is around 60% but in one review the rate of live births was 92.3% [5, 9].

As a conclusion, it is important to emphasize the need for systematic exploration of the pelvis upon the first ultrasound scan of the pregnancy performed at 7-8 weeks of gestation, even if an intrauterine gestational sac is already confirmed and even if there is no apparent risk factor. It is a fact that the diagnosis of HP tends to be overlooked after confirming the intrauterine pregnancy. Index of suspicion must be higher when the patient has undergone IVF or if tubal damage is suspected. When a diagnosis is established on time, the rate of pregnancies that reach term after treatment is significant. Finally, it seems important to limit the number of embryos transferred, according to the guidelines established by international committees for assisted reproductive technology.

## References

[1] Basile F, Cesare CD, Quagliozzi L (2012). Spontaneous heterotopic pregnancy, simultaneous ovarian, and intrauterine: a case report. *Case Reports in Obstetrics and Gynecology*.

[2] Govindarajan MJ, Rajan R (2008). Heterotopic pregnancy in natural conception. *Journal of Human Reproductive Sciences*.

[3] Selo-Ojeme DO, GoodFellow CF (2002). Simultaneous intrauterine and ovarian pregnancy following treatment with clomiphene citrate. *Archives of Gynecology and Obstetrics*.

[4] Kiely JL, Kiely M (2001). Epidemiological trends in multiple births in the United States, 1971–1998. *Twin Research*.

[5] Divry V, Hadj S, Bordes A, Genod A, Salle B (2007). Case of progressive intrauterine twin pregnancy after surgical treatment of cornual pregnancy. *Fertility and Sterility*.

[6] Inion I, Gerris J, Joostens M, de Vree B, Kockx M, Verdonk P (1998). An unexpected triplet heterotopic replacement of two embryos. *Human Reproduction*.

[7] Nikolaou DS, Lavery S, Bevan R, Margara R, Trew G (2002). Triplet heterotopic pregnancy with an intrauterine monochorionic diamniotic twin pregnancy and an interstitial pregnancy following in vitro fertilisation and transfer of two embryos. *Journal of Obstetrics and Gynaecology*.

[8] Cholkeri-Singh A, LaBarge A (2007). Spontaneous heterotopic triplets: a case report. *Fertility and Sterility*.

[9] Bugatto F, Quintero-Prado R, Kirk-Grohar J, Melero-Jiménez V, Hervías-Vivancos B, Bartha JL (2010). Heterotopic triplets: tubal ectopic and twin intrauterine pregnancy. A review of obstetric outcomes with a case report. *Archives of Gynecology and Obstetrics*.

[10] Sun SY, Júnior EA, Júnior JE (2012). Diagnosis of heterotopic pregnancy using ultrasound and magnetic resonance imaging in the first trimester of pregnancy: a case report. *Case Reports in Radiology*.

[11] Tal J, Haddad S, Gordon N, Timor-Tritsch I (1996). Heterotopic pregnancy after ovulation induction and assisted reproductive technologies: a literature review from 1971 to 1993. *Fertility and Sterility*.

[12] Jackson H, Granger S, Price R (2008). Diagnosis and laparoscopic treatment of surgical diseases during pregnancy: an evidence-based review. *Surgical Endoscopy and Other Interventional Techniques*.

[13] Reedy MB, Källén B, Kuehl TJ (1997). Laparoscopy during pregnancy: a study of five fetal outcome parameters with use of the Swedish Health Registry. *American Journal of Obstetrics and Gynecology*.

[14] Al-Fozan H, Tulandi T (2002). Safety and risks of laparoscopy in pregnancy. *Current Opinion in Obstetrics and Gynecology*.

[15] Okamura Y, Arakane F, Nagayoshi-Taura Y, Honda R, Ohba T, Katabuchi H (2011). Heterotopic triplet pregnancy: report of a patient with remnant tubal ectopic and intrauterine twin pregnancy after frozen-thawed embryo transfer. *Reproductive Medicine and Biology*.

[16] Goldstein JS, Ratts VS, Philpott T, Dahan MH (2006). Risk of surgery after use of potassium chloride for treatment of tubal heterotopic pregnancy. *Obstetrics and Gynecology*.

